# Global variability in dental unit waterline safety standards: bridging the gap between guidelines and practice

**DOI:** 10.3389/froh.2026.1896545

**Published:** 2026-10-14

**Authors:** Kimia Sadat Kazemi, Yalda Anvari, Michelle F. Siqueira

**Affiliations:** College of Dentistry, University of Saskatchewan, Saskatoon, SK, Canada

**Keywords:** antimicrobial resistance, antimicrobial stewardship, biofilm formation, dental infection control, dental unit waterlines, water quality standards

## Abstract

**Background:**

Dental unit waterlines (DUWLs) are a recognized yet frequently underappreciated source of microbial contamination in dental environments. Their narrow tubing, intermittent water flow, and internal surface characteristics create favorable conditions for rapid biofilm growth. These biofilms can persist and release microorganisms into the water used during dental procedures, potentially increasing the risk of infection.

**Methods:**

This review synthesizes global guidelines, regulatory frameworks, and emerging evidence related to DUWL contamination, with particular attention to regional variations in standards, compliance expectations, and monitoring practices.

**Results:**

Although most guidelines converge on potable-water quality for nonsurgical procedures and sterile water or saline for surgical care, substantial international variation persists in implementation and enforcement. North America generally provides relatively consistent guidance, though adherence remains uneven, while Europe and the Asia–Pacific region exhibit marked differences in microbial thresholds, surveillance practices, and regulatory oversight. Beyond bacterial contamination, emerging evidence highlights the clinical relevance of fungal colonization and the potential role of DUWL biofilms as reservoirs for antimicrobial resistance. These issues remain insufficiently addressed in many regulatory systems. Inadequately controlled DUWL contamination can contribute to preventable infections, inflammatory complications, and diagnostic uncertainty, which may lead to unnecessary antibiotic prescriptions. This highlights the importance of incorporating environmental infection control into dentistry's antimicrobial stewardship efforts. This review also explores the persistent gap between knowledge and practice among dental professionals. It identifies opportunities for future advancements, including artificial intelligence–assisted monitoring, nanotechnology-enhanced antimicrobial materials, and environmentally sustainable disinfection strategies.

**Conclusions:**

Overall, the findings underscore the need for harmonized international guidance supported by enforceable monitoring standards and adaptable implementation pathways for resource-limited settings. Strengthening DUWL management through evidence-based protocols, technological innovation, and enhanced professional education is essential to reducing contamination risks and promoting safe, high-quality dental care globally.

## 1 Introduction

Water plays an essential role in dental operatory procedures, supplying coolant for rotating instruments and irrigation for the operative field; however, despite its routine use, the microbiological environment within dental unit waterlines (DUWLs) remains an often-overlooked source of potential contamination (1–5). DUWLs are composed of narrow-bore polymer tubing often no wider than 2–3 mm, creating confined environments characterized by low flow rates, intermittent use, stagnation, and high surface-to-volume ratios (1, 6–11). These conditions promote rapid microbial colonization and biofilm establishment, with measurable formation occurring within a few days, effectively converting DUWLs into a persistent and complex microbial ecosystem (12–19).

DUWLs function as conduits that deliver water from municipal sources or independent reservoir systems to handpieces, air–water syringes, and ultrasonic scalers within the dental operatory (20–23). The aforementioned physical design and usage patterns create an ideal setting for microbial adhesion and persistence (1, 24, 25). Microorganisms may be introduced through incoming water supplies, contaminated reservoir bottles, or oral backflow during clinical procedures, particularly when anti-retraction mechanisms are absent, malfunctioning, or inadequately maintained (3, 13, 26).

Once microorganisms adhere to the inner surfaces of DUWL tubing, they proliferate and form structured biofilms along the tubing walls (21, 27, 28). Consistent with established biofilm developmental models, these communities progress from initial attachment to microcolony formation and extracellular polymeric matrix production within a short time frame (21, 24). Encased within this protective matrix, microorganisms exhibit enhanced resistance to environmental stressors and disinfection efforts, allowing biofilms to persist despite routine water flow (14, 29). These biofilms are more than passive microbial accumulations (2, 12, 30). They serve as continuous reservoirs capable of releasing microorganisms into the water stream during routine dental procedures (12, 31–33). As water is activated through handpieces, ultrasonic scalers, or air–water syringes, bacteria and other microorganisms may be dislodged, enter the water stream, and flow either directly into the oral cavity or dispersed into the surrounding environment via aerosols and fine splatter (34–38).

Sheltered within their matrix, microorganisms such as *Legionella pneumophila*, *Pseudomonas aeruginosa*, nontuberculous *Mycobacteria*, and fungal species survive, adapt, and persist within DUWLs (23, 26, 30, 39, 40). In healthy individuals, this exposure will likely be unnoticed. However, immunocompromised individuals exposed to contaminated DUWLs have been linked to severe infections, including Legionnaires' disease (12, 30, 41, 42). Documented outbreaks include cases of hospitalization and death (43) and show DUWLs being an active participant in the chain of infection, with serious health implications (13, 23, 34).

The ability of biofilms to develop into structured microbial communities with defined spatial organization and their persistence underscores the clinical relevance of DUWL contamination and highlights its importance within the broader context of infection control in dental settings (6, 33, 35, 36, 38, 44).

Attempts to control these microorganisms reveal the resilience of biofilms. Microorganisms are protected within a matrix that resists eradication, making simple flushing ineffective (1, 4, 37, 45). Effective management, therefore, requires regular flushing, chemical disinfection, applied continuously or intermittently, and continuous microbiological monitoring (1, 46). Yet adherence to these measures remains inconsistent, with nearly one-third of dental professionals reporting limited awareness of DUWL guidelines, constrained by time, cost, training gaps, and the variability of dental unit designs (12, 43, 47, 48).

Differences in regulations across locales, combined with emerging concerns such as fungal contamination and antimicrobial resistance, make it difficult to establish unified standards (12, 49, 50). The COVID-19 pandemic increased global attention on aerosols and airborne transmission within clinical environments and brought renewed urgency to questions related to infection control (43). These factors underscore the need for unified, evidence-based guidelines that consider both existing and emerging risks in dental unit water systems. Evidence indicates that gaps in compliance with guidelines remain (34, 51–53), and that new disinfection technologies and monitoring approaches may change how DUWLs are managed (34, 54, 55). As expectations for safety continue to grow, the need for contemporary, evidence-informed protocols becomes increasingly clear (10, 34, 50, 56). This review synthesizes the path of water through DUWLs, examining how unseen processes shape patient safety and how informed interventions can improve safety in routine elements of dental care. This review critically examines global variability in DUWL safety standards, contamination risks, compliance challenges, and emerging approaches to water quality management and infection prevention.

## 2 Historical background, advances, and emerging technologies

The issue of DUWL contamination emerged in the 1960s with water-cooling systems for high-speed handpieces, replacing early air-cooling designs (57, 58). Studies from the 1980s first identified biofilm formation in DUWLs (11, 12, 28, 50), showing the tendency for these systems to support persistent microbial communities. Subsequent outbreaks of Legionella in the 1990s highlighted the clinical risks of DUWL contamination, leading to the development of infection control guidelines and risk management strategies in dental practices (13, 59). Technological advances in the 1990s included self-contained reservoirs for treated water (1, 20), and advances in the 2000s included anti-retraction valves/inline microfilters to reduce backflow and microbial load (1, 20, 21, 28). In 2003, the U.S. Centers for Disease Control and Prevention (CDC) issued comprehensive guidelines establishing a microbial quality threshold of ≤500 CFU/mL for water used in nonsurgical dental procedures (1, 10, 15) (Figure 1).

**Figure 1:**
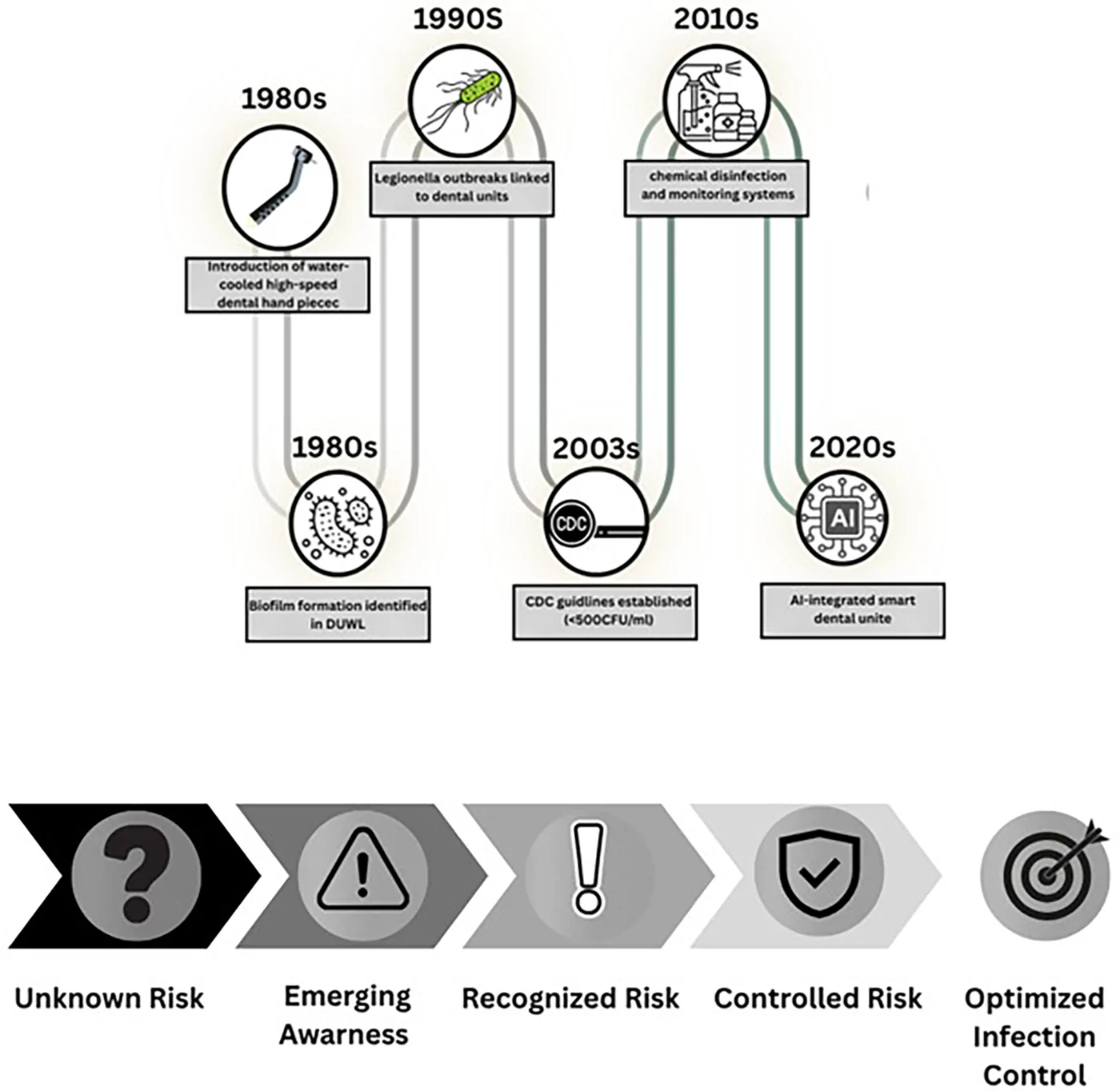
Timeline highlighting key milestones in the understanding and management of DUWL contamination, including the introduction of water-cooled handpieces in the 1960s, identification of biofilms in the 1980s, legionella-associated outbreaks in the 1990s, establishment of CDC water quality standards in 2003, and the subsequent development of continuous disinfection technologies.

These guidelines also emphasized the need for continuous waterline disinfection, using agents such as hydrogen peroxide or silver ion–based treatments, and the use of automated flushing devices.

Pathogenesis in DUWLs is driven by the development of biofilm, which forms under conditions of water stagnation, reduced flow rates, backflow, and the inherent ability of microorganisms to adhere to the internal surfaces of the tubing (12, 60) (Figure 2). Risk factors include immunocompromised patients, contaminated source water, and suboptimal waterline maintenance (1, 23). Prevention includes flushing the waterlines for 2–3 min at the start of each day and for 20–30 s between patients, chemical disinfectants (chlorine, iodine), and sterile surgical irritants (42). Emerging technologies like AI for real-time microbial monitoring via sensors and nanotechnology (e.g., silver nanoparticle tubing coatings) offer sustained antimicrobial effects (12, 61). Future solutions may combine AI-driven predictive maintenance and nanotechnology to eliminate biofilms, pending research on scalability and cost (1).

**Figure 2:**
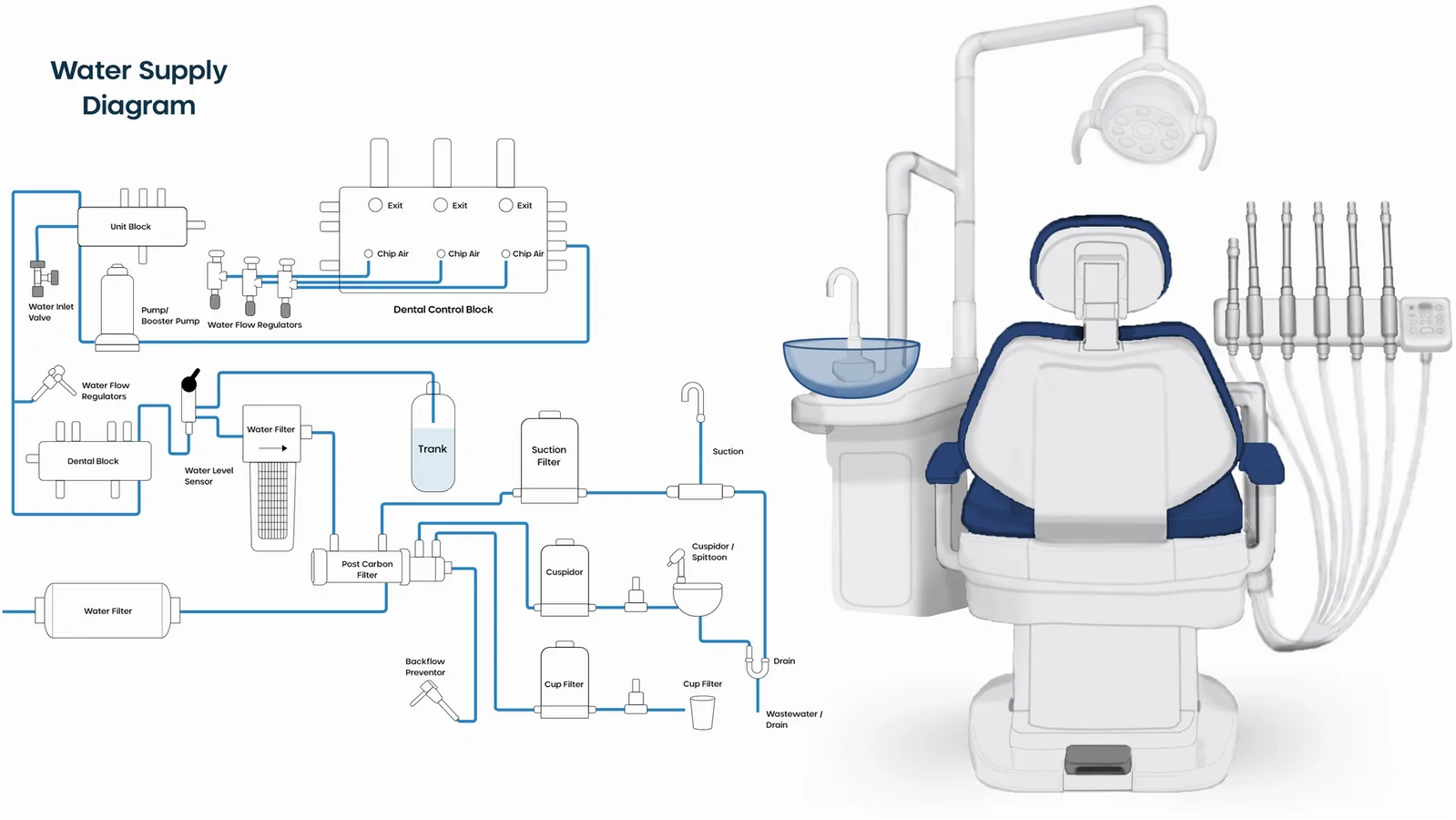
Schematic representation of a DUWL system, illustrating the main components and water flow pathways alongside a standard dental operatory unit. The diagram highlights key elements including the water supply, filtration system, control block, and delivery to handpieces and auxiliary outlets. This figure is intended for illustrative purposes only; configurations and components may vary depending on the manufacturer, model, and age of the dental unit.

Recent evidence indicates that a substantial proportion of DUWLs samples worldwide fail to meet recommended microbial quality standards. Non-compliance reports vary significantly across studies, reflecting differences in geographic region, sampling methods, monitoring frequency, and maintenance practices, with microbial counts frequently exceeding 500 CFU/mL despite existing preventive measures (47, 57, 61, 62). Such contamination not only threatens patient safety but also imposes substantial economic and legal burdens, including treatment costs for healthcare-associated infections, clinic closures, and reputational damage to dental practices (63). Although several reviews have addressed DUWL contamination and control strategies, comparatively few have synthesized guideline compliance patterns across settings alongside the post-COVID emphasis on aerosol risk**.** In the post-COVID era, when aerosol-generating procedures have received renewed scrutiny, DUWL water quality has become even more central to infection prevention. This review, therefore, aims to critically synthesize current standards, summarize reported compliance challenges, and appraise emerging innovations to inform more harmonized international protocols (47, 64–66). Recent advances (2020s) highlight emerging applications of artificial intelligence and nanotechnology for real-time monitoring and long-term biofilm prevention (47, 67, 68).

Regardless of the source water used, municipal, distilled, or bottled, microorganisms may be introduced into DUWLs via the water supply itself or through backflow of patient saliva or blood. In the confined environment of narrow-bore tubing, biofilms rapidly develop and may contain bacteria such as *Legionella pneumophila*, *Pseudomonas aeruginosa*, nontuberculous mycobacteria, and fungal species. These biofilms act as persistent reservoirs, continuously shedding microorganisms that are disseminated in aerosols during routine dental procedures (3, 47, 49, 69) (Figure 3).

**Figure 3:**
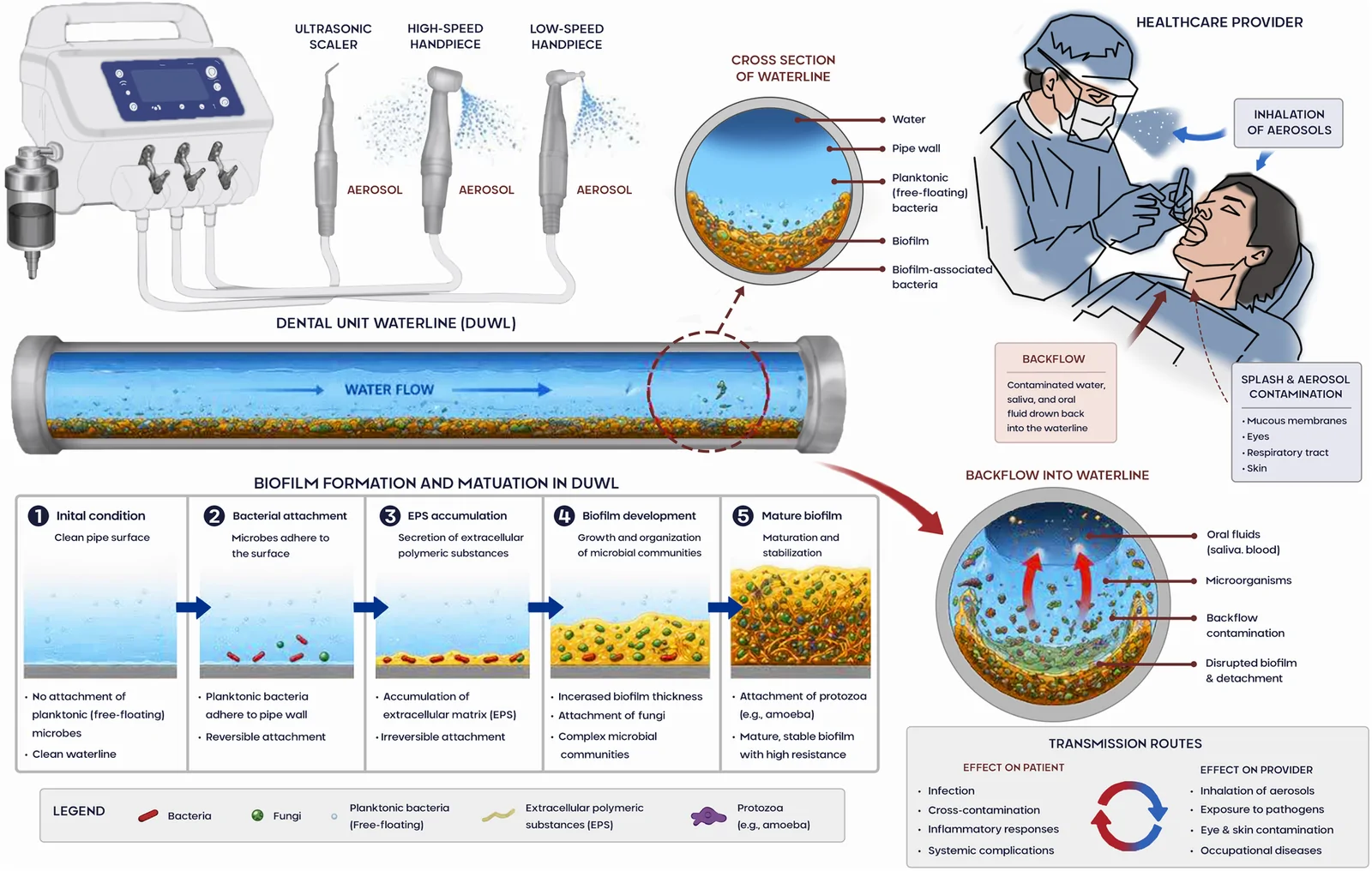
Schematic illustration of biofilm formation and microbial contamination in DUWLs, demonstrating the progression from initial bacterial attachment to mature biofilm development. The figure highlights key processes including extracellular polymeric substance (EPS) production, biofilm maturation, and detachment, as well as the role of dental instruments (e.g., ultrasonic scaler, high- and low-speed handpieces) in generating aerosols. Potential routes of transmission to patients and healthcare providers are depicted, including inhalation of aerosols, splatter contamination, and backflow into waterlines. This figure is intended for illustrative purposes only; actual conditions and configurations may vary depending on clinical settings, equipment, and maintenance practices.

## 3 Methods

This narrative literature review was conducted to synthesize current scientific evidence together with official regulatory and professional guidance related to DUWL cleaning, maintenance, water quality, and infection control.

To ensure comprehensive coverage of contemporary literature, a structured search was performed in PubMed, Scopus, and Web of Science. The structured database search focused on publications from January 2020 through November 2025 to identify recent peer-reviewed evidence, contemporary guidelines, emerging developments, research gaps, and areas of consensus related to DUWL management. The structured database search was designed to identify contemporary evidence and was not intended to represent the sole source of evidence included in this narrative review. The complete database-specific search strategies, including database-specific search syntax, Boolean operators, and the date of the final search for each database, are provided in Supplementary Table S1.

The date restriction applied only to the structured electronic database search and was not intended to exclude seminal publications, landmark studies, foundational guideline documents, or historically important evidence. In keeping with the narrative nature of this review, the structured database search was complemented by manual reference checking, citation tracking, and targeted searches of official governmental agencies, regulatory authorities, professional organizations, and public health institutions. These complementary approaches were used to identify original guideline documents, historically important studies, and regulatory resources that continue to underpin current recommendations but may predate the structured search period or may not be indexed in conventional bibliographic databases.

Whenever appropriate, original landmark publications and primary guideline documents were cited directly rather than through secondary references to accurately reflect the evolution of current concepts and recommendations.

**Figures:** All figures were created by the authors for illustrative purposes using *Adobe Illustrator* (version 28.0; Adobe Inc., San Jose, CA, USA), *Adobe Photoshop* (version 25.0; Adobe Inc.), and *Canva* (Canva Pty Ltd., Sydney, Australia). No previously published figures, copyrighted images, screenshots, or graphical materials were reproduced. The authors retain the original editable source files.

### 3.1 Literature search and evidence sources

#### 3.1.1 Evidence selection

Official Evidence was selected according to its relevance to the objectives of this narrative review. Priority was given to:

- Official guidelines, standards, recommendations, and position statements issued by international, national, or regional governmental agencies, regulatory authorities, and professional organizations (e.g., CDC, ADA, CDA, WHO, APSIC, RCDSO, and HTM 01-05);
- Peer-reviewed experimental, observational, and clinical studies investigating DUWL contamination, water quality, cleaning protocols, disinfection strategies, monitoring methods, or compliance with recommended standards;
- Review articles and expert commentaries that contributed to the interpretation of current evidence or provided clinically relevant perspectives on DUWL management.

To maximize the international scope of the review, official regulatory documents and guidance from multiple countries were considered. Whenever available, official English-language guidance documents were preferentially used. Where complete guidance documents were available only in the official language of the respective country, the original documents were reviewed by the authors, and the sections relevant to DUWL management were translated by the authors for data extraction and synthesis. No formal study selection or critical appraisal was performed because this review was narrative in nature.

Sources outside the scope of this review generally included publications not directly related to DUWL cleaning, water quality, or infection prevention, as well as conference abstracts without an accessible full-text publication.

Reference lists of key publications were manually reviewed, where appropriate, to identify additional relevant evidence.

#### 3.1.2 Data synthesis

The collected evidence was synthesized narratively according to geographic region (United States, Canada, Europe, and Asia-Pacific) and thematic areas, including water quality standards, flushing protocols, chemical treatment strategies, monitoring practices, and regulatory requirements.

## 4 Guidelines by region

### 4.1 United States

#### 4.1.1 National guidelines and water quality standards

In the United States, no single federal regulation governs DUWLs. Instead, guidelines from the Centers for Disease Control and Prevention (CDC) and the American Dental Association (ADA) define the national standard of care, which most states have incorporated into their dental practice regulations. In states that have not formally codified these guidelines, compliance is still enforced by state dental boards through licensure requirements, inspections, and disciplinary mechanisms (45, 47, 70–72).

These CDC and the ADA guidelines align with the United States Environmental Protection Agency (EPA) (73) drinking water standards for dental treatment water. Accordingly, the recommended water quality target for nonsurgical dental procedures is ≤500 colony-forming units per milliliter (CFU/mL) of heterotrophic bacteria. For surgical procedures, a higher level of asepsis is required, and sterile saline or sterile water must be used, delivered through sterile tubing systems or appropriate bypass devices (47, 72, 74).

#### 4.1.2 Flushing protocols

Recommended flushing protocols for DUWLs include flushing the system for 2–3 min at the start of each clinical day to reduce overnight microbial accumulation. In addition, flushing for 20–30 s between patients is advised to decrease the planktonic microbial load within the waterlines, although this practice does not eliminate established biofilms (47, 72, 75).

#### 4.1.3 Treatment and disinfection

The use of EPA-registered disinfectants approved for DUWL treatment is strongly recommended as part of routine maintenance protocols (76). These include continuous chemical germicides delivered through the water system, as well as tablets or cartridges designed for use in independent water bottle systems. In addition, periodic “shock” treatments are advised to control and disrupt established biofilms within the waterlines, thereby enhancing the overall effectiveness of disinfection strategies (47, 72).

#### 4.1.4 Monitoring and documentation

Routine microbial monitoring (commonly quarterly) is recommended to verify compliance with the standard of ≤500 CFU/mL. Results should be documented and retained as part of the infection prevention program (34, 47, 72).

#### 4.1.5 State-specific DUWL requirements

While the CDC and ADA provide national guidance on DUWL management, regulatory enforcement occurs primarily at the state dental board level. California, New York, Texas, Florida, Massachusetts, and Illinois were selected as representative examples because their publicly available regulations or guidance documents explicitly address DUWL maintenance, documentation, monitoring, or compliance with CDC infection control recommendations. This list is illustrative rather than exhaustive, as requirements vary across states and are not always explicitly detailed in publicly accessible regulatory documents.

##### 4.1.5.1 California

DUWL management is regulated by the California Dental Board, which mandates strict adherence to CDC Guidelines for Infection Control in Dental Health-Care Settings (47). DUWL water quality must not exceed ≤500 colony-forming units per milliliter (CFU/mL), consistent with potable water standards (47). The use of EPA-registered disinfectants is required for routine waterline treatment. In addition, dental practices are obligated to maintain detailed records of maintenance and monitoring activities, which must be readily available for regulatory inspection. Failure to comply with these requirements may result in disciplinary action. Consequently, California is widely recognized as one of the most stringent U.S. states in enforcing infection control standards in dental settings (73, 77).

##### 4.1.5.2 New York

Dental infection control practices are regulated under the authority of the New York State Education Department, which requires adherence to CDC infection control guidelines. Dental practices must establish and maintain a written infection control program outlining standard procedures and responsibilities (47). Specific to DUWLs, maintenance and flushing protocols must be clearly documented as part of routine infection control measures. Additionally, for all surgical procedures, the use of sterile water or saline is mandated to ensure aseptic conditions and minimize the risk of cross-contamination (77, 78).

##### 4.1.5.3 Texas

Dental infection control practices are overseen by the Texas State Board of Dental Examiners, which expects compliance with CDC and the ADA recommendations. Maintenance of DUWLs is considered part of the minimum standard of care in clinical practice (47). Emphasis is placed on the use of anti-retraction devices to prevent backflow contamination and reduce the risk of cross-infection. Failure to adhere to these standards may be interpreted as professional negligence and could have legal implications, including potential malpractice claims (72, 75, 79).

##### 4.1.5.4 Florida

Dental infection control practices are regulated by the Florida Board of Dentistry, which mandates the implementation of CDC infection control guidelines. DUWLs must be routinely treated and monitored to ensure compliance with recommended water quality standards (47). Comprehensive documentation of maintenance and monitoring activities is required and must be available during regulatory inspections. Particular emphasis is placed on aerosol-generating procedures, given their potential role in the transmission of infectious agents within dental settings (74, 75).

##### 4.1.5.5 Massachusetts

Dental infection control practices are regulated by the Massachusetts Board of Registration in Dentistry, which explicitly requires DUWL water quality to be maintained at ≤500 colony-forming units per milliliter (CFU/mL), consistent with established potable water standards (47). DUWL management is incorporated into routine office infection control audits to ensure compliance. The use of chemical treatment systems is recommended as part of standard protocols to control microbial contamination and prevent biofilm formation within the waterlines (75, 80).

##### 4.1.5.6 Illinois

Dental infection control practices fall under the authority of the Illinois Department of Financial and Professional Regulation, where compliance with CDC guidelines is considered a professional obligation. Particular emphasis is placed on routine monitoring of DUWL water quality to ensure adherence to recommended standards (47). In addition, dental practices are required to maintain proper documentation of DUWL maintenance activities as part of their infection control protocols and for regulatory oversight (75, 81).

### 4.2 Canada

In Canada, DUWL management is governed by provincial infection prevention and control guidelines issued by dental regulatory authorities and professional associations (82).

These guidelines generally align with the CDC recommendation of ≤500 colony-forming units per milliliter (CFU/mL) for dental treatment water. Routine flushing protocols typically include 2–3 min at the start of each day and 20–30 s between patients, although slight variations exist across provinces (e.g., Alberta recommends 2 min daily and 20 s post-care). Waterline treatment commonly involves daily enzymatic cleaning and periodic (e.g., weekly) disinfection, with recommendations to avoid the use of water heaters that may promote microbial growth. Regular water quality monitoring is advised, particularly for the management of vulnerable patient populations, along with the implementation of contingency plans during water advisories. The use of sterile water or saline is required for surgical procedures. Overall, Canadian guidelines emphasize routine flushing, manufacturer-directed disinfection, ongoing monitoring, and preparedness for water-related public health concerns (47, 82–85).

#### 4.2.1 DUWL guidance by province/region

##### 4.2.1.1 British Columbia (BC)

In British Columbia, the BC College of Oral Health Professionals (BCCOHP) provides guidance on DUWL management through provincial infection prevention and control (IPC) standards. DUWL management is addressed as a dedicated component within the provincial infection prevention and control (IPAC) guidelines, including requirements for maintenance, treatment, and monitoring in accordance with manufacturer instructions and the clinic's infection control program (86).

##### 4.2.1.2 Alberta (AB)

The College of Dental Surgeons of Alberta (CDSA) and other Alberta dental regulatory authorities provide clear guidance on DUWL management. Flushing is recommended for a minimum of 2 min at the start of each clinical day and at least 20 s after each patient. Water quality is expected to meet potable drinking water standards, effectively aligning with the ≤500 CFU/mL threshold. Additionally, the use of DUWL heaters is discouraged, as elevated temperatures may promote microbial proliferation and biofilm formation (84).

##### 4.2.1.3 Saskatchewan (SK)

The College of Dental Surgeons of Saskatchewan (CDSS) provides guidance on DUWL management within the broader framework of infection prevention and control. For systems using municipal water, flushing is recommended for at least 2 min at the start of each clinical day, consistent with provincial IPC standards. In addition, structured monitoring and testing programs, such as periodic or annual DUWL water quality assessment, are referenced as part of comprehensive quality assurance and infection control protocols (87).

##### 4.2.1.4 Manitoba (MB)

The Manitoba Dental Association (MDA), in conjunction with provincial infection prevention and control (IPC) resources, provides guidance on DUWL management with a strong emphasis on disinfection practices. DUWLs should be disinfected strictly in accordance with manufacturer instructions and established standard operating procedures. Additionally, specific guidance is provided for source-water contingencies, such as during boil-water advisories, where enhanced precautions are recommended, including flushing incoming waterlines and implementing supplementary infection control measures to ensure patient and provider safety (85).

##### 4.2.1.5 Ontario (ON)

In Ontario, the Royal College of Dental Surgeons of Ontario (RCDSO), in alignment with Public Health Ontario, provides comprehensive guidance on IPC in dental settings. The core approach emphasizes that dental practices are responsible for maintaining, monitoring, and regularly updating IPC protocols, including those related to water quality management. RCDSO guidance documents also include specific considerations for DUWLs, such as routine maintenance, implementation of appropriate treatment and monitoring procedures, and the avoidance of waterline heaters due to their potential to promote microbial growth and biofilm formation. In addition, dental practices are required to establish formal DUWL maintenance policies to support consistent monitoring, documentation, and compliance with infection prevention and control standards (88, 89).

##### 4.2.1.6 Québec (QC)

In Québec, the Ordre des dentistes du Québec (ODQ) provides guidance on infection prevention and control (IPC) through its document *Prévention et contrôle des infections*. This guidance addresses the management of contamination risks in dental settings, including those associated with dental water systems and DUWLs, through the implementation of procedural controls and adherence to recommended practices. In addition, earlier ODQ documentation highlights operational considerations related to water conduits and the management of alternative water sources during municipal water contamination events or public health advisories (90).

##### 4.2.1.7 New Brunswick (NB)

In New Brunswick, the New Brunswick Dental Society (NBDS), in collaboration with provincial regulators, provides guidance on IPC in dental settings. The revised 2020 IPC guideline includes explicit recommendations for DUWL management, emphasizing disinfection in accordance with manufacturer instructions and outlining procedures to be followed during boil-water advisories. More recently, the introduction of an updated IPAC standard in 2025 has strengthened the regulatory framework by establishing infection prevention requirements as enforceable minimum standards, with non-compliance considered professional misconduct (91).

##### 4.2.1.8 Nova Scotia (NS)

In Nova Scotia, the Nova Scotia Regulator of Dentistry and Dental Assisting (NSRDDA), in collaboration with the Nova Scotia Dental Association, provides comprehensive oversight of infection prevention and control (IPAC) in dental settings. The Standard of Practice mandates compliance with provincial IPAC requirements, including defined frameworks for enforcement, inspection, and professional accountability. Earlier guidance documents include specific recommendations for DUWL management, such as routine maintenance, avoidance of waterline heaters, and the use of monitoring systems to ensure water quality. More recently, harmonized IPAC standards published between 2022 and 2025 by provincial regulators have further strengthened and standardized these requirements across the province (92–96).

##### 4.2.1.9 Prince Edward Island (PEI)

In Prince Edward Island, the Dental Association of Prince Edward Island requires compliance with IPC guidelines issued by the association. However, while these guidelines are referenced in publicly available materials, specific requirements related to DUWL management are not explicitly detailed in accessible documents. Therefore, based on currently available information, PEI guidance can be described as referencing general IPC standards without providing publicly detailed DUWL-specific requirements (97, 98).

##### 4.2.1.10 Newfoundland and Labrador (NL)

In Newfoundland and Labrador, the Newfoundland and Labrador Dental Board, together with provincial dental hygiene regulators, provides guidance on infection prevention and control in oral healthcare settings. Professional standards outline expectations for IPC compliance, including evaluation and inspection frameworks, while official IPC policies and procedures provide guidance applicable to oral health care providers (99).

### 4.3 Europe

Across Europe, DUWL management is governed by heterogeneous national standards rather than a unified EU guideline, with most countries applying potable water and Legionella control regulations to dental settings (100). As a result, DUWL water quality thresholds, disinfection practices, and monitoring requirements vary substantially between countries, ranging from strict microbiological limits and mandatory testing (e.g., Germany) to largely advisory or inconsistently implemented practices (101–103).

#### 4.3.1 DUWL guidance by country

##### 4.3.1.1 United Kingdom (UK)

In the United Kingdom, the National Health Service (NHS) provides guidance on DUWL management through HTM 01-05, *Decontamination in Primary Care Dental Practices*. This guidance emphasizes maintaining water quality at potable standards, with particular focus on the control of *Legionella*. Flushing is recommended for 20–30 s between patients, as well as at the start and end of each clinical day. Chemical disinfection is advised as a primary treatment strategy, and the use of independent bottle systems is common in practice. In addition, monitoring is based on risk assessment for *Legionella*, supported by periodic microbiological testing to ensure water safety (104–107).

##### 4.3.1.2 Germany

In Germany, the microbiological quality of DUWLs is governed by both the current German Drinking Water Ordinance (Trinkwasserverordnung, TrinkwV) and the recommendations of the Commission for Hospital Hygiene and Infection Prevention (KRINKO) at the Robert Koch Institute. Rather than prescribing specific disinfectants or standardized treatment concentrations, the German framework establishes microbiological performance criteria, including a total bacterial count of <100 CFU/mL and Legionella concentrations of <100 CFU/100 mL. Potable-quality water is required for routine dental procedures, whereas sterile water should be used for surgical interventions. Regular microbiological monitoring, typically performed at least annually, is recommended, and corrective decontamination measures should be implemented whenever microbiological thresholds are exceeded. The selection of disinfectants and treatment protocols is generally based on validated manufacturer-specific instructions rather than universally prescribed chemical regimens (25, 108).

##### 4.3.1.3 France

In France, DUWL management is guided by public health and dental regulatory bodies, with defined microbiological benchmarks for both inlet and outlet water. Inlet water is expected to contain <100 CFU/mL, while outlet water should not exceed a 10-fold increase relative to the inlet. Sterile water is mandatory for surgical procedures. In practice, approximately 65% of dental clinics perform daily flushing (typically 20–30 s), and around 62% utilize chemical disinfectants for waterline treatment. However, routine microbiological monitoring remains limited, with only about 2.6% of practices conducting regular testing (109).

##### 4.3.1.4 Netherlands

In the Netherlands, the National Institute for Public Health and the Environment (RIVM) provide guidance on DUWL management, with a strong emphasis on *Legionella* control. Potable water standards are applied to dental units to ensure baseline water quality. Rather than routine DUWL-specific testing, a risk-based monitoring approach is recommended, focusing on system assessment and targeted evaluation to mitigate potential contamination risk (103, 110, 111).

##### 4.3.1.5 Belgium

In this context, no dedicated guideline exists specifically for DUWL management. Water quality is instead governed by general drinking water legislation, and decisions regarding DUWL disinfection practices are largely left to professional judgment within the clinical setting (112, 113).

#### 4.3.2 Scandinavian countries

##### 4.3.2.1 Sweden

In this context, strong national drinking water standards form the basis of water quality regulation, while infection prevention and control guidance places particular emphasis on the prevention of *Legionella*. However, specific thresholds for DUWL quality are not explicitly defined, leaving aspects of DUWL management to be guided by general water safety principles (103, 114).

##### 4.3.1.2 Norway

Healthcare water systems are regulated under the potable water and Legionella frameworks.

DUWL maintenance guided by manufacturer instructions (115, 116).

##### 4.3.1.3 Denmark

In this context, there is a strong emphasis on the microbial safety of water used in healthcare settings. However, no separate regulation exists specifically for DUWLs, and chemical treatment is commonly employed in practice as a primary strategy for maintaining water quality and controlling microbial contamination (117, 118).

##### 4.3.1.4 Switzerland

Although not part of the European Union, this country is often included in European comparative analyses of DUWL management. It applies strict potable water quality standards, with *Legionella* thresholds also extended to dental water systems. Periodic monitoring is encouraged to ensure compliance and maintain microbiological safety (119, 120).

##### 4.3.1.5 Eastern & Southern Europe

In many Southern and Eastern European countries, no DUWL-specific national standards have been identified; instead, water safety in dental settings is largely governed by general drinking water regulations and Legionella control legislation, with monitoring practices (121) often limited or inconsistently implemented (122).

##### 4.3.1.6 Spain

Spanish regulatory documents related to Legionella prevention are published primarily in Spanish and were therefore summarized descriptively in the Supplementary Material rather than included in the evidence synthesis (123, 124).

### 4.4 Asia-Pacific

Across the Asia–Pacific region, DUWL management is guided primarily by Asia Pacific Society of Infection Control (APSIC) and CDC-aligned infection control principles rather than DUWL-specific national regulations, while allowing flexibility for resource-limited settings. Emphasis on potable water standards (≤500 CFU/mL), flushing, chemical disinfection, and staff training (61). While countries such as Australia have established explicit, enforceable recommendations, including routine monitoring and shock disinfection, many others rely on general drinking water standards, Legionella control frameworks, and manufacturer-recommended protocols, resulting in heterogeneous implementation across the region (61, 64).

#### 4.4.1 DUWL guidance by country/region

##### 4.4.1.1 Australia

In Australia, the Australian Dental Association (ADA), in collaboration with state health departments, provides clear and accessible guidance on DUWL management. Water quality is recommended to meet ≤500 CFU/mL, with sterile water required for surgical procedures. Flushing protocols include at least 2 min at the start of the day and 20–30 s between patients, with guidance to avoid the use of warm water due to its potential to promote microbial growth. Continuous chemical disinfection is advised, supplemented by periodic shock treatments. In addition, the ADA recommends monthly water quality testing and proper documentation to ensure ongoing compliance and patient safety (125) (Table 1).

**Table 1:** Overview of regulatory frameworks, professional guidance, and compliance mechanisms governing DUWL management across selected regions and countries.

Region/Country	Regulatory authority	Water quality standard	Flushing protocols	Treatment & disinfection	Monitoring & enforcement	References
Countries and regions with established DUWL regulations or enforceable professional guidance						
United States (national)	CDC; ADA (state-level enforcement)	≤500 CFU/mL (nonsurgical); sterile water/saline for surgery	2–3 min at start of day; 20–30 s between patients	EPA-registered chemical disinfectants; continuous systems; periodic shock treatments	Routine microbial monitoring (often quarterly); documentation required; enforced by state dental boards	(47)
California (USA)	California Dental Board	≤500 CFU/mL	Per CDC	EPA-registered disinfectants mandatory	Mandatory documentation, inspections, and disciplinary action for non-compliance	(126)
New York (USA)	NY State Education Department	≤500 CFU/mL; sterile water for surgery	Per CDC	CDC-compliant disinfection	Written infection control program; documentation required	(127)
Texas (USA)	Texas State Board of Dental Examiners	CDC/ADA-aligned	Per CDC	Emphasis on anti-retraction devices; disinfection per guidance	Considered part of the minimum standard of care; malpractice risk	(128)
Florida (USA)	Florida Board of Dentistry	CDC-aligned	Per CDC	Regular treatment required	Documentation reviewed during inspections	(129)
Massachusetts (USA)	MA Board of Registration in Dentistry	≤500 CFU/mL	Per CDC	Chemical treatment systems are recommended	Included in office infection control audits	(130)
Canada (national pattern)	Provincial dental regulators	≤500 CFU/mL (CDC-aligned)	2–3 min daily; 20–30 s between patients	Enzymatic/chemical disinfection; avoid water heaters	Monitoring emphasized for vulnerable patients; provincial enforcement	(82)
British Columbia	BCCOHP	Manufacturer-directed (CDC-aligned)	As per IPC program	Manufacturer-directed treatment	Required as part of clinic IPC program	(86)
Alberta	CDSA	Potable water benchmark (≈≤500 CFU/mL)	2 min daily; 20 s post-patient	Disinfection per guidance; avoid heaters	Documented within IPC policies	(131)
Ontario	RCDSO	CDC-aligned	Per IPC guidance	Maintenance & monitoring emphasized	Mandatory maintenance policies	(89)
Saskatchewan(SK)	College of Dental Surgeons of Saskatchewan	Not explicitly stated (aligned with national standards)	∼2 min at start of day (municipal supply)	Not strictly specified; integrated within IPC protocols	Structured monitoring recommended (e.g., periodic/annual DUWL testing) as part of quality assurance frameworks	(132)
Manitoba(MB)	Manitoba Dental Association (MDA)/provincial IPC resources	Not explicitly stated (aligned with national standards)	Not strictly specified; adapted during advisories	Disinfection strictly according to manufacturer instructions and SOPs	Additional precautions during water advisories (e.g., boil-water advisories), including flushing and supplementary measures	(85)
Québec (QC)	Ordre des dentistes du Québec (ODQ)	Not explicitly stated (aligned with national standards)	Not specifically defined	IPC guidelines emphasize procedural controls and adherence to best practices for managing DUWL contamination	Includes guidance on managing contamination risks and alternative water sources during municipal advisories	(90)
New Brunswick (NB)	New Brunswick Dental Society/provincial regulators	Not explicitly stated (aligned with national standards)	Not specifically defined	Disinfection according to manufacturer instructions; IPC guideline revised in 2020 includes DUWL management and procedures during boil-water advisories	Updated 2025 IPAC standard establishes enforceable minimum requirements; non-compliance considered professional misconduct	(91)
Germany	Trinkwasserverordnung (GDWO)	<100 CFU/mL; Legionella <100 CFU/100 mL	Defined in water safety plans	Mandatory disinfection if exceeded	At least annual testing	(25, 108)
France	National public health authorities	Inlet <100 CFU/mL; outlet ≤10× inlet	Daily flushing (20–30 s)	Chemical disinfectants common	Monitoring rare (∼2.6%)	(109)
Countries and regions without DUWL-specific regulations and reliance on general drinking-water standards						
Europe (general)	National public health authorities	Variable; potable water + Legionella laws	Variable	Variable	Highly heterogeneous; no unified EU DUWL standard	(100)
United Kingdom	NHS (HTM 01-05)	Potable water standard	20–30 s between patients; daily flushing	Chemical disinfection; independent bottle systems	Risk-based Legionella assessment	(107)
Netherlands	RIVM	Potable water standard	Risk-based	Legionella-focused control	Risk-based monitoring	(110)
Scandinavia (SE, NO, DK)	National public health agencies	Potable water standards	Not DUWL-specific	Legionella-focused; manufacturer-guided	Limited DUWL-specific monitoring	(114, 115, 117)
Switzerland	Federal Office of Public Health	Strict potable water standards	Risk-based	Legionella thresholds applied	Periodic monitoring encouraged	(119)
Southern & Eastern Europe	National/regional authorities	Drinking water + Legionella laws	Variable	Manufacturer-guided	Monitoring limited or inconsistent	(100)
Asia–Pacific (general)	APSIC; national bodies	≤500 CFU/mL (CDC-aligned); sterile for surgery	2 min daily; 20 s between patients	Chemical germicides; shock treatments	Variable; training emphasized	(61)
Australia	Australian Dental Association	≤500 CFU/mL; sterile for surgery	≥2 min daily; 20–30 s between patients	Continuous disinfection; shock treatments	Monthly testing recommended	(125)
Other APAC countries	National health authorities	Potable water standards	Variable	Manufacturer/CDC-aligned	Inconsistent; resource-dependent	(61, 133)

The table summarizes water quality standards, recommended flushing protocols, treatment and disinfection strategies, and monitoring or enforcement practices in jurisdictions with established DUWL regulations, as well as regions where DUWL management relies primarily on general drinking-water standards or Legionella-focused risk assessments.

### 4.5 Comparison of chemical disinfection strategies across international guidelines

Although chemical disinfection is universally recognized as an essential component of DUWL maintenance, substantial variability exists among national and regional guidance documents regarding disinfectant selection, treatment protocols, and implementation strategies. Most regulatory authorities do not prescribe a universal disinfectant concentration or contact time. Instead, they establish microbiological performance targets and recommend that dental practitioners use validated DUWL treatment systems according to manufacturers' instructions (45, 47, 72).

In the United States, the CDC, the ADA, and most state dental boards emphasize maintaining DUWL output water at ≤500 CFU/mL while recommending EPA-registered or validated treatment systems without specifying standardized disinfectant concentrations (45, 47, 72). Similarly, Canadian provincial regulators—including Ontario, Alberta, Saskatchewan, Manitoba, British Columbia, and New Brunswick, primarily focus on microbiological water quality, routine flushing protocols, periodic monitoring, and adherence to manufacturers' instructions, rather than mandating specific chemical formulations or exposure times (84–87, 89–91, 93, 99).

European guidance follows a comparable performance-based approach but demonstrates greater heterogeneity in implementation (103). The United Kingdom recommends validated treatment systems together with flushing protocols and risk-based Legionella management, whereas Germany emphasizes microbiological monitoring and corrective actions through RKI/KRINKO guidance without specifying universal disinfectant concentrations (103, 105, 134–137) (Table 2).

**Table 2:** Comparative summary of chemical disinfection and maintenance recommendations for DUWLs across selected national, state, provincial, and regional guidelines.

Guideline/Country	Disinfectant named	Universal concentration specified?	Continuous treatment	Shock treatment	Monitoring	Manufacturer instructions	Legionella surveillance	References
CDC (USA)	EPA-registered products	No	✓	✓	Routine microbial monitoring	✓	Recognized as a potential pathogen; target species not specified	(47)
California	Not specified	No	✓	Product-dependent	✓	✓	Not specified	(126)
Washington	Validated DUWL products	No	✓	✓	Required	✓	Not specified	(138)
New York	Not specified	No	✓	Product-dependent	✓	✓	Not specified	(127)
Texas	Not specified	No	✓	Product-dependent	✓	✓	Not specified	(47)
Ontario	Not specified	No	✓	If indicated	✓	✓	Not specified; IPAC checklist focuses on water quality, biofilm, treatment methods, training, flushing, and monitoring	(89)
Alberta	Not specified	No	✓	If indicated	✓	✓	Not specified; DUWL section included, but no specific distinction between *L. pneumophila* and *Legionella* spp. found in the guideline	(84)
Saskatchewan	Not specified	No	✓	Corrective disinfection	Annual	✓	Not specified; annual DUWL testing and corrective disinfection if >500 CFU/mL, but no *Legionella* species target specified	(87)
Manitoba	Not specified	No	✓	✓	Routine testing	✓	*Legionella* species mentioned as organisms isolated from DUWLs, but no specific provincial testing target identified	(85)
British Columbia	Not specified	No	✓	If indicated	✓	✓	Not specified; DUWL biofilm microorganisms described generally as bacteria, fungi, and protozoa	(86)
New Brunswick	Not specified	No	✓	If indicated	✓	✓	Not specified; DUWL section follows general waterline maintenance approach	(91)
Québec	Not specified	No	✓	If indicated	IPC	✓	Not specified	(90)
United Kingdom	Validated products	No	✓	✓	Legionella risk assessment	✓	**Legionella** (risk assessment required; species not specified in HTM guidance)	(107)
Germany	No universal agent	No	✓	Corrective disinfection	Annual microbiology	✓	**Legionella spp.** (RKI guidance follows microbiological monitoring for Legionella in process water; not restricted to *L. pneumophila*)	(108)
France	Chemical systems	No	✓	✓	Periodic	✓	Not specified in dental guidance (Legionella monitoring discussed, species generally not specified)	(109)
Netherlands	Not specified	No	✓	If indicated	Legionella monitoring	✓	**Legionella spp.** (Legionella control programs rather than *L. pneumophila* only)	(110)
Belgium	Not specified	No	✓	If indicated	IPC	✓	Not specified	(112)
Switzerland	Not specified	No	✓	If indicated	Periodic	✓	Not specified	(119)
Spain	Not specified	No	✓	If indicated	IPC	✓	Not specified	(123)
Scandinavia	Not specified	No	✓	If indicated	Legionella-focused	✓	Not specified	(115, 117, 119)

This table summarizes water-quality targets, flushing protocols, disinfectant recommendations, treatment strategies, monitoring requirements, and reliance on manufacturer instructions. Only information explicitly stated in official regulatory or professional guidance documents was extracted; where disinfectants, concentrations, or contact times were not specified, this was recorded as “not specified” rather than inferred. DUWL, infection prevention and control.

Despite considerable variation in regulatory frameworks, a consistent pattern emerged across international guidance documents. Nearly all regulatory authorities emphasize achieving microbiologically safe dental unit water rather than prescribing universally applicable disinfectant concentrations or exposure times. Water-quality targets, routine flushing, microbiological monitoring, and biofilm control constitute the core recommendations worldwide. In contrast, the selection of disinfectants, treatment concentrations, contact times, and maintenance intervals is generally delegated to validated manufacturer-specific protocols. This approach reflects the wide variability in dental unit designs, integrated water treatment technologies, and commercially available DUWL treatment systems. Consequently, concentrations reported in experimental studies should not be interpreted as regulatory requirements unless they are explicitly incorporated into official guidance documents.

Therefore, the present review distinguishes regulatory recommendations from experimental evidence and reports only those disinfectants, concentrations, and treatment protocols that are explicitly stated in official guidance documents. Where guidance documents defer these parameters to manufacturers' instructions, this has been indicated accordingly in Table 2.

International guidance also differs in the reporting of *Legionella* surveillance. While some European guidance documents refer broadly to *Legionella spp.* or require a *Legionella* risk assessment (107), most national and regional dental guidelines do not explicitly specify whether surveillance should target *Legionella pneumophila* or all *Legionella* species. To avoid overinterpretation, the present review reports only the terminology explicitly stated in the original guidance documents.

A notable finding of the present review was that most regulatory authorities do not prescribe specific disinfectants, concentrations, or contact times. Instead, they recommend the use of validated DUWL treatment products and defer detailed treatment protocols to manufacturers' instructions for use. Consequently, disinfectant concentrations frequently reported in laboratory studies (e.g., hydrogen peroxide- or chlorine dioxide-based protocols) should not be interpreted as regulatory requirements unless explicitly incorporated into official guidance documents (Table 3).

**Table 3:** Comparison of disinfectants reported in official national guidelines and manufacturer instructions for use (IFUs) for dental unit waterline (DUWL) maintenance.

Disinfectant	Mentioned in national guideline?	Mentioned in manufacturer IFU?	Evidence source	References
Hydrogen peroxide	Rarely	✓	Experimental + IFU	(139)
Chlorine dioxide	Rarely	✓	Experimental + IFU	(140)
Sodium hypochlorite	Rarely	✓	IFU	(141)
Silver-based systems	Rarely	✓	IFU	(142)
Iodine-based systems	Rarely	✓	IFU	(143)

This table illustrates the distinction between regulatory recommendations and manufacturer-specific maintenance protocols. While most national and regional guidelines emphasize microbiological water-quality targets and recommend the use of validated DUWL treatment systems, they rarely specify individual disinfectants, standardized concentrations, or contact times. In contrast, manufacturer instructions for use (IFUs) commonly provide detailed information on disinfectant selection, dilution protocols, contact or dwell times, and maintenance procedures. Evidence source indicates whether the recommendation originates primarily from official guidance documents, manufacturer IFUs, or experimental studies.

This distinction is particularly important because several laboratory studies report effective disinfectant concentrations under controlled experimental conditions, whereas regulatory authorities generally focus on microbiological performance outcomes and defer operational treatment protocols to validated manufacturer-specific instructions.

Manufacturer instructions provide substantially greater operational detail than most national or regional guidelines. Among the manufacturers reviewed, A-dec provides a “Maintain–Monitor–Shock” framework, combining continuous ICX maintenance with periodic ICX Renew shock treatment; the publicly available IFU specifies 2.1% hydrogen peroxide for ICX Renew Solution 1 and a dwell time of 12–96 h (144). KaVo provides explicit hydrogen peroxide concentrations for OXYGENAL 6, with approximately 0.02% H₂O₂ during routine continuous germ reduction and 0.25% H_2_O_2_ during intensive germ reduction (144, 145). Dentsply Sirona describes an integrated disinfection system using Dentosept, automatic purge/auto-purge programs, and periodic sanitation, with manufacturer guidance indicating that Dentosept should remain in the waterlines for at least 24 h during intensive sanitation (146). Planmeca describes an automated hydrogen peroxide–based waterline cleaning system based on weekly overnight treatment, while Midmark emphasizes daily flushing, continuous treatment, regular monitoring, and shock treatment when heterotrophic bacterial counts exceed the desired level (147, 148). These examples illustrate those disinfectant concentrations, dwell times, and treatment frequencies are usually defined at the level of the manufacturer-specific system rather than by national regulation (Table 4).

**Table 4:** Comparison of manufacturer-specific recommendations for dental unit waterline (DUWL) maintenance and chemical disinfection protocols.

Manufacturer	Continuous treatment	Shock/sanitization	Disinfectant/active agent	Concentration	Contact/dwell time	Monitoring/notes	Reference
A-dec	ICX tablets	ICX Renew shock	Hydrogen peroxide-based shock solution; ICX tablets for maintenance	ICX Renew Solution 1: 2.1% H_2_O_2_	12–96 h	Maintain–Monitor–Shock approach; monthly testing initially, then every 3 months if stable	A-dec IFU/Waterline guide (144)
KaVo	Integrated continuous germ reduction	Intensive germ reduction	OXYGENAL 6; hydrogen peroxide with silver ions	**0.02% H_2_O_2_** during routine operation; **0.25% H_2_O_2_** during intensive germ reduction	Product/program-specific	Automatic dosing through KaVo unit	KaVo OXYGENAL 6 (145)
Dentsply Sirona	Integrated disinfection system	Monthly sanitization/after prolonged non-use	Dentosept disinfectant	Not publicly specified as universal concentration	≥24 h Dentosept retention for optimal biofilm protection	Automatic flushing, purge/auto-purge, sanitation programs	Dentsply Sirona infection-prevention guidance (146)
Planmeca	System-dependent; no continuous H₂O₂ exposure in some systems	Weekly overnight waterline cleaning	Hydrogen peroxide-based Planmeca waterline cleaning system	Not publicly specified as universal concentration	Overnight treatment	Automated waterline cleaning; no continuous H₂O₂ exposure to patient/team in described system	Planmeca Waterline Cleaning System (148)
Midmark	Continuous treatment recommended	Shock treatment if heterotrophic bacteria level is high	Product-dependent; often third-party validated DUWL products	Not specified; follow product IFU	Product-specific	Four elements: flushing, continuous treatment, monitoring, shock if needed	Midmark Waterline Maintenance (147)

This table summarizes the publicly available instructions for use (IFUs) of major dental unit manufacturers regarding continuous treatment, shock disinfection, disinfectant type, reported concentrations, contact/dwell times, and monitoring recommendations. Only concentrations and treatment parameters explicitly stated in official manufacturer documentation are reported. The table highlights the distinction between manufacturer-specific operational protocols and national regulatory guidance, as most regulations define microbiological performance targets while referring clinicians to validated manufacturer instructions for disinfectant selection, concentration, contact time, and maintenance procedures.

## 5 Discussion

### 5.1 Regional variability in standards, compliance, and implementation

This review confirms that DUWLs remain a persistent and globally relevant infection control challenge. Although most contemporary guidelines converge on the use of potable water standards (≤500 CFU/mL) for nonsurgical dental procedures and mandate sterile water or saline for surgical care, substantial heterogeneity exists in how these standards are implemented, monitored, and enforced across regions (47).

In North America, particularly the United States and Canada, DUWL management frameworks are strongly shaped by CDC, ADA, and Canadian professional guidance (47, 72, 82). These recommendations are widely accepted as the national standard of care and are operationalized through state- or province-level regulatory mechanisms. However, despite the clarity of these standards, compliance remains inconsistent. Financial constraints, aging infrastructure, limited access to water testing services, and variable staff training have been repeatedly identified as barriers to sustained compliance (3, 49, 149). This disconnect between well-defined standards and real-world implementation illustrates that regulatory clarity is insufficient without investment in education, infrastructure, and accountability systems (150).

In contrast, Europe demonstrates marked regulatory heterogeneity and monitoring practices. Certain countries, most notably Germany, enforce substantially stricter microbiological thresholds (<100 CFU/mL total bacterial count and <100 CFU/100 mL for Legionella) under national drinking water legislation (108). These findings highlight regional differences in both microbiological standards and surveillance practices. Survey-based studies from France indicate that routine microbial monitoring of DUWLs is rarely performed, with only approximately 2.6% of dental practices reporting regular testing (151). This finding highlights a critical gap between regulatory ambition and operational feasibility, suggesting that stringent standards without enforceable monitoring frameworks may have limited practical impact.

The Asia–Pacific region illustrates an additional layer of complexity. APSIC-aligned recommendations broadly mirror CDC principles, emphasizing potable water standards, routine flushing, chemical disinfection, and staff education (47, 61). Nevertheless, implementation is highly variable, particularly in resource-limited settings where access to disinfectants, independent water bottle systems, and microbiological testing is inconsistent. In several countries, DUWL maintenance relies heavily on manufacturer-recommended protocols rather than nationally enforced regulations, increasing variability in both compliance and effectiveness. These challenges are compounded by disparities in professional training and awareness regarding biofilm formation and long-term DUWL risks.

Despite regional differences, there are commonalities. For example, flushing protocols and the conceptual adoption of potable water standards are widely shared across regions. However, Europe and the Asia–Pacific demonstrate greater variability in enforcement and lower reported compliance compared with North America, as exemplified by minimal routine monitoring in France and resource-driven constraints in parts of Asia (47, 61). These international contrasts are summarized in Table 1, which illustrates how similar guidelines yield divergent outcomes depending on regulatory structure, enforcement mechanisms, and healthcare infrastructure.

Although several experimental studies have evaluated intensive shock-disinfection protocols, such as the application of chlorine concentrations of approximately 20 mg/L every three days combined with routine flushing, may be effective in disrupting established biofilms (1, 100). However, the long-term feasibility, DUWL material compatibility, and occupational safety implications of such regimens remain insufficiently studied. Furthermore, fungal contamination of DUWLs, which is infrequently addressed in existing guidelines, represents an underrecognized risk, particularly for immunocompromised patients (62). Persistent knowledge gaps among dental professionals regarding biofilm ecology and DUWL maintenance further exacerbate these issues.

Collectively, these findings highlight a fundamental limitation of current international DUWL management: the absence of harmonized guidance supported by standardized monitoring and enforceable implementation strategies. While regional guidelines provide valuable frameworks, their effectiveness is ultimately constrained by inconsistent implementation, limited surveillance, and variable professional awareness (3, 47). Harmonized international recommendations, integrating evidence-based microbiological thresholds, feasible monitoring strategies, and adaptable implementation pathways for resource-limited settings, are urgently needed. Without such coordination, DUWL contamination will likely remain a preventable yet persistent source of infection risk in dental healthcare worldwide (23).

An important distinction identified in this review is the disconnect between experimental evidence evaluating disinfectant efficacy and the recommendations contained within national regulatory guidance. Laboratory and clinical studies have demonstrated the effectiveness of various disinfectants, including hydrogen peroxide, chlorine dioxide, sodium hypochlorite, and silver-based formulations, under controlled conditions. However, these findings have not been uniformly translated into national regulations. Instead, most guidelines emphasize achieving microbiological water-quality targets while recommending the use of validated disinfectants according to manufacturer instructions, rather than prescribing universal disinfectant concentrations or standardized treatment protocols. Consequently, considerable variation exists among countries regarding the choice of disinfectants, maintenance schedules, and the use of continuous treatment vs. periodic shock disinfection. This regulatory flexibility likely reflects differences in dental unit design, compatibility of disinfectants with equipment materials, availability of commercial treatment systems, and national regulatory frameworks. More broadly, this review demonstrates that many recommendations currently implemented worldwide continue to rely on expert consensus and manufacturer guidance rather than high-quality comparative clinical evidence. Addressing this evidence gap should be a priority for future research through multicentre prospective studies evaluating the effectiveness, feasibility, and cost-effectiveness of DUWL maintenance protocols across diverse clinical environments. Such evidence will be essential to support the development of internationally harmonized, evidence-based recommendations and facilitate more consistent implementation of best practices.

An equally important consideration is the feasibility of implementing DUWL quality assurance strategies in routine clinical practice. While some proposed solutions rely on specialized equipment or costly infrastructure that may not be practical for widespread adoption, scalable approaches based on routine monitoring, evidence-based maintenance protocols, and continuous quality assurance can be readily implemented across academic institutions, community clinics, and private dental practices. These practical strategies not only facilitate broader implementation but also generate standardized multicentre data that can support benchmarking, strengthen the evidence base, and ultimately inform future policy development and harmonization of international guidelines. Ultimately, the greatest impact is likely to come from practical, evidence-based strategies that are affordable, reproducible, and easily integrated into routine dental practice, allowing research findings to translate more rapidly into improvements in patient safety, clinical care, and educational practice.

### 5.2 Beyond bacteria: emerging microbial and resistance-related threats in DUWLs within a one health and antimicrobial stewardship framework

While the majority of existing guidelines and surveillance frameworks for DUWLs focus almost exclusively on bacterial contamination, accumulating evidence indicates that DUWL biofilms represent far more complex and clinically relevant microbial ecosystems. In addition to well-established bacterial pathogens, fungi such as Candida albicans and Aspergillus fumigatus are increasingly identified within DUWL biofilms and have been implicated in opportunistic infections, particularly among immunocompromised patients (150, 152–156). The major microorganisms detected in DUWL biofilms and their associated clinical risks are summarized in Table 5, highlighting the breadth of potential health implications for both patients and dental professionals.

**Table 5:** Major microorganisms identified in DUWL biofilms and their clinical relevance.

Microorganism	Type	Source	Associated disease/risk	Notes
Legionella pneumophila	Bacterium	Municipal water	Legionnaires' disease, pneumonia	Aerosol transmission documented in dental clinics (23, 135, 160, 161)
Pseudomonas aeruginosa	Bacterium	Environmental biofilms	Opportunistic infections, otitis, respiratory illness	Highly resistant to disinfectants (161)
Nontuberculous mycobacteria (NTM)	Bacterium	Waterline biofilm	Pulmonary and skin infections	Difficult to eradicate; forms thick biofilm (161)
Candida albicans	Fungus	Biofilm/patient backflow	Oral or systemic candidiasis	Increasing post-COVID concern (14, 154)
Aspergillus fumigatus	Fungus	Airborne contamination	Allergic bronchopulmonary aspergillosis	Resilient spores persist in tubing (14)

Another area of variation identified among international recommendations is *Legionella* surveillance. While *Legionella* is widely recognized as one of the principal opportunistic waterborne pathogens associated with DUWLs, guidance documents differ considerably in the level of detail provided for monitoring. Some recommendations refer broadly to *Legionella* spp. or require a *Legionella* risk assessment, whereas others do not specify the target organism or analytical approach. This lack of consistency reflects differences in national surveillance strategies, regulatory priorities, and risk assessment frameworks. Harmonizing recommendations for *Legionella* surveillance may improve comparability between monitoring programs and facilitate more standardized approaches to DUWL risk management internationally.

The clinical significance of DUWL contamination extends beyond microbial water-quality indicators, as documented cases demonstrate that contaminated dental water can result in clinically significant infections. Outbreaks of *Mycobacterium abscessus* following pediatric pulpotomy procedures have resulted in cervical lymphadenitis, mandibular and maxillary osteomyelitis, pulmonary nodules, hospitalization, surgical intervention, prolonged antimicrobial treatment, and permanent tooth loss. Severe infection has also been documented among immunocompetent children, indicating that the clinical risk is not necessarily restricted to medically vulnerable populations (157).

In addition, *Legionella pneumophila* pneumonia associated with exposure to a contaminated dental unit waterline has been reported, illustrating the potential for DUWL-associated exposure to extend beyond localized odontogenic infection (43, 158).

From a mechanistic perspective, DUWL biofilms act as persistent microbial reservoirs from which microorganisms may be released into treatment water, exposing patients through direct contact, ingestion, or aerosol inhalation. Importantly, investigations of reported infections have identified heavily contaminated water systems and deficiencies in waterline maintenance and monitoring. Collectively, these findings emphasize that bridging the gap between existing infection-control recommendations and their consistent implementation is not merely a matter of achieving microbiological water-quality targets but an essential component of patient safety (3).

DUWL biofilms should be viewed through a One Health framework because they exist at the intersection of environmental water systems, the built healthcare environment, microbial ecology, and human health. Rather than representing an isolated dental infection control issue, DUWL contamination illustrates the interconnected nature of environmental, clinical, and healthcare-associated microbial ecosystems. Water supplied to dental units originates from municipal distribution systems and enters a complex built environment characterized by narrow-bore tubing, intermittent water flow, variable temperatures, and extensive internal surfaces that promote microbial attachment and biofilm maturation. Once established, these biofilms develop into dynamic microbial communities composed of bacteria, fungi, protozoa, and other microorganisms that continuously interact with one another and with their surrounding environment. Consequently, DUWLs should not be regarded as passive reservoirs of contamination but as active microbial ecosystems capable of influencing water quality, microbial ecology, the emergence and persistence of antimicrobial resistance, and ultimately patient safety. This perspective reinforces the importance of integrating infection prevention, environmental monitoring, and antimicrobial stewardship within a unified One Health approach (21, 161).

Consistent with this ecological perspective, numerous studies have demonstrated that DUWL biofilms harbor opportunistic waterborne microorganisms, particularly Legionella spp., which are frequently detected using culture-based and molecular diagnostic approaches (69, 159). From a One Health perspective, fragmented and inconsistent DUWL regulations not only undermine patient safety but also hinder coordinated responses to environmental antimicrobial resistance, underscoring the need for integrated international guidance that simultaneously addresses environmental reservoirs and clinical risk. Beyond their role in harboring pathogens, DUWL biofilms can also serve as reservoirs of antimicrobial resistance (AMR) determinants (162, 163). The dense, multispecies architecture of biofilms can support horizontal gene transfer, creating conditions that promote the emergence and persistence of resistant microorganisms (164–166). This is a particularly important concern in the context of the global escalation of AMR, yet existing DUWL guidelines rarely incorporate surveillance or strategies that target AMR (163). Repeated exposure to sublethal concentrations of disinfectants may select for microorganisms that are more tolerant or resistant, further supporting the role of DUWLs as environmental reservoirs of resistance (163, 165). During routine dental procedures, aerosolization of water from DUWLs can allow microorganisms to spread beyond the patient, increasing exposure risks for dental personnel and contributing to contamination of the clinical environment (34). Despite these risks, current monitoring practices remain largely confined to heterotrophic bacterial counts, which does not capture these additional concerns (13). Applying a One Health framework broadens the objectives of DUWL surveillance beyond conventional bacterial water-quality testing. Future monitoring strategies should integrate environmental microbiology, fungal contamination, antimicrobial resistance markers, occupational exposure, and standardized reporting systems. Such an approach would facilitate collaboration among dental professionals, microbiologists, environmental scientists, manufacturers, and public health authorities, ultimately strengthening infection prevention and antimicrobial stewardship (167, 168). Expanding DUWL surveillance to include fungal contamination and resistance-related markers would help to strengthen infection control measures within a One Health-oriented approach (163, 168, 169).

Evidence from different jurisdictions further illustrates the distinction between the existence of infection-control recommendations and their effective implementation. In California, the 2016 Orange County outbreak of invasive nontuberculous mycobacterial infections following pediatric dental procedures revealed important deficiencies in DUWL disinfection and routine monitoring and subsequently contributed to strengthened state requirements for water used during procedures involving exposed dental pulp (170).

A similar outbreak occurred in Georgia, where children developed *Mycobacterium abscessus* infections following pulpotomy procedures. The investigation demonstrated the absence of routine water-quality monitoring and appropriate waterline disinfection, while all sampled dental stations exceeded the recommended microbial threshold; molecular typing further supported the dental-unit water as the source of infection (158).

Evidence from Italy additionally demonstrates that DUWL-associated clinical consequences are not limited to odontogenic mycobacterial infections, as *Legionella pneumophila* pneumonia associated with a contaminated dental unit waterline has been reported (43).

Collectively, these examples demonstrate that failures in DUWL maintenance and monitoring can have clinically significant consequences across different settings. Nevertheless, they primarily represent outbreak investigations and individual clinical events rather than systematic population-level surveillance (135). Consequently, although appropriate implementation of DUWL guidelines is associated with improved microbiological water quality, the available evidence remains insufficient to establish a quantitative correlation between the stringency of regulations and the prevalence of DUWL-associated clinical infections across jurisdictions. This limitation highlights the need for standardized reporting and surveillance of suspected and confirmed DUWL-associated infections (158).

### 5.3 The knowledge-practice gap in DUWL management

Despite the availability of evidence-based recommendations from international bodies such as the CDC, ADA, and APSIC, real-world implementation of DUWL protocols remains suboptimal. Multiple studies indicate that up to one-third of dental professionals are either unaware of or unable to consistently implement recommended DUWL maintenance procedures due to financial constraints, inadequate training, time pressures, and variability in dental unit design. Although the COVID-19 pandemic heightened global awareness of aerosol-mediated transmission risks, this increased concern has not translated into sustained or systematic improvements in DUWL management practices (43, 47, 61, 171–173).

Daily flushing is the most adopted practice, with reported compliance rates approaching 70% (42, 47, 100). In contrast, more resource-intensive or knowledge-dependent measures, such as continuous disinfection, routine microbial monitoring, staff training, and maintenance of written DUWL protocols, demonstrate markedly lower compliance, ranging between 25% and 55% (42). This persistent knowledge–practice gap underscores the limitations of guideline-based approaches when there are insufficient enforcement, auditing, and educational reinforcement. Without stronger regulatory oversight and institutional accountability, DUWL guidelines may not be consistently incorporated into clinical practice (174, 175) (Table 6).

**Table 6:** Matrix of potential factors contributing to the gap between DUWL recommendations and their implementation in dental practice.

Major contributing factor	Mechanism contributing to the gap	Potential impact if unaddressed	(28, 176)
Knowledge and training gaps	Limited understanding of treatment, testing, interpretation, or corrective procedures	Incorrect or inconsistent DUWL maintenance and delayed corrective action	(28, 176)
Time and staffing constraints	Monitoring, treatment and documentation compete with routine clinical duties	Irregular maintenance and testing	(176)
Financial/resource limitations	Recurrent costs of testing, disinfectants, equipment maintenance and staff training	Reduced monitoring frequency or incomplete implementation	(176)
Unclear responsibility	No designated staff member responsible for DUWL management	Missed, inconsistent, or undocumented procedures	(176)
Inconsistent microbial monitoring	Treatment may be performed without verification of water quality	Persistent contamination or treatment failure may remain undetected	(28, 176)
Unclear response to failed tests	Practices may lack predetermined corrective protocols	Continued use of contaminated units and delayed remediation	(176)
Variation/complexity of protocols	Recommendations may differ by jurisdiction, product, or manufacturer	Confusion and inconsistent implementation	(176)
Limited regulatory oversight	Monitoring and compliance may not be routinely audited or enforced	Greater variability between recommended and actual practice	(176)
Biofilm persistence/DUWL design	Narrow tubing, stagnation and biofilm favor microbial persistence and recolonization	Recurrent contamination despite intermittent treatment	(3, 176)

### 5.4 Future directions: artificial intelligence, nanotechnology, and smart systems

Emerging technologies offer promising solutions to longstanding challenges in DUWL safety. Artificial intelligence-enabled systems, integrated with real-time microbial sensors, may improve how DUWLs are managed by allowing earlier detection rather than reacting when contamination occurs (177). Such systems could enable continuous detection of microbial contamination, predictive modeling of biofilm development, and automation of optimal flushing and disinfection schedules (178). Integration with electronic dental records may further allow automated compliance logging, helping to facilitate audits, accreditation processes, and quality assurance (179).

In parallel, nanotechnology developments increase the potential for passive, long-term biofilm control approaches. Nanoparticle-based coatings incorporating silver, copper, or zinc demonstrate sustained antimicrobial activity and may reduce reliance on frequent chemical disinfection methods. Photodynamic disinfection, using light-activated photosensitizers, represents another eco-friendly approach that can target biofilms with minimal chemical residue. A synthesis of these innovations is presented in Table 7, which outlines their principles, advantages, and current limitations (178, 180, 181).

**Table 7:** Emerging technologies and innovations for DUWL safety.

Innovation	Principle/technology	Advantages	Challenges/limitations	Status/future direction
AI-based microbial sensors	Real-time detection via biosensors	Predictive maintenance; automation	Cost, calibration, data privacy	Prototype testing (2020s) (182)
Nanoparticle coatings	Silver, copper, zinc nanofilms	Long-term antimicrobial action	Scalability; potential cytotoxicity	Experimental; promising (182)
Photodynamic disinfection	Light-activated photosensitizers	Eco-friendly, biofilm-targeted	Requires energy input; wavelength limits	Early research stage (183)
Smart dental units	Integrated monitoring & auto-logging	Enhances compliance and traceability	High installation cost	Under development (15)
Eco-friendly agents	Plant-based antimicrobials, enzymes	Sustainable and safe	Less evidence for efficacy	Pilot studies (15)

While these approaches are promising, several challenges remain. High initial costs, scalability, long-term material safety, and limited clinical validation currently constrain widespread adoption (184). Likely, a hybrid strategy that combines AI-driven predictive maintenance, nanotechnology-enhanced materials, and environmentally sustainable disinfectants could represent an effective management strategy for DUWL systems. However, clinical trials and cost–benefit analyses will be essential to determine the feasibility of translating these innovations into routine practice (184).

### 5.5 Policy implications and the need for global harmonization

A fundamental barrier to progress in DUWL safety is the absence of harmonized international standards. As demonstrated throughout this review, regional variability in microbiological thresholds, monitoring requirements, and enforcement mechanisms generates confusion among dental professionals, complicates multicenter research, and perpetuates inequities in patient safety. The current patchwork of national and regional guidelines, ranging from enforceable regulatory frameworks to largely advisory recommendations, undermines the effectiveness of infection prevention efforts (Figure 4).

**Figure 4:**
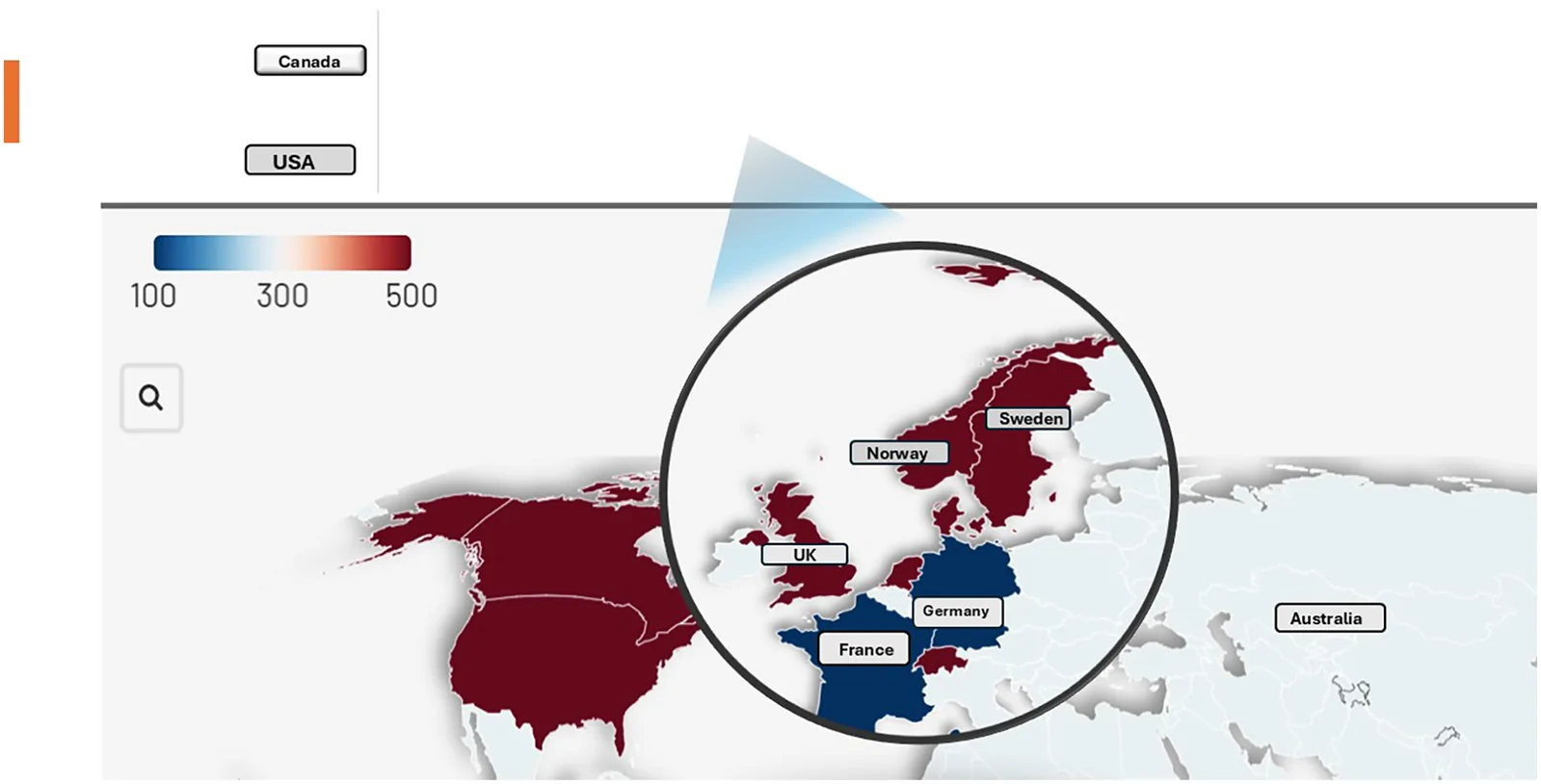
The absence of harmonized international DUWL standards leads to variable compliance worldwide. National and regional guidelines (CDC/ADA, RCDSO, EU, APSIC) adopt different microbial thresholds (expressed as CFU/mL) and monitoring frequencies. A unified WHO- or FDI-coordinated framework could standardize these thresholds, ensure mandatory testing, and strengthen global patient safety.

Strengthening global DUWL safety would benefit from an internationally coordinated framework that establishes consistent microbiological thresholds, routine monitoring requirements, and standardized training and certification protocols. Beyond harmonized benchmarks, improvements could include the development of uniform terminology and reporting standards, manufacturer obligations for validated waterline performance and post-market surveillance, and engineering innovations that minimize biofilm formation through improved tubing materials, automated flushing mechanisms, and systems designed to reduce stagnation. A structured approach to documentation, including treatment logs, water-quality test records, and maintenance schedules, would enhance traceability and support regulatory oversight.

Additionally, integrating DUWL management principles into undergraduate dental curricula and continuing professional development would reinforce practitioner competence and long-term adherence. Together, these elements would promote a more consistent, evidence-based approach to DUWL safety across diverse clinical and regulatory environments.

Current DUWL guidelines primarily focus on microbiological water quality and biofilm control, while environmental aspects of wastewater disposal receive limited attention. Following DUWL disinfection, residual disinfectants are typically discharged into municipal wastewater systems; however, specific recommendations regarding acceptable residual concentrations or disposal procedures are rarely provided in dental-specific guidance and are generally addressed through broader environmental regulations. Future guidelines should better integrate infection prevention with environmentally responsible wastewater management.

A practical pathway toward international DUWL surveillance and guideline harmonization could involve a stepwise approach. Initially, a core set of minimum indicators could be established, including microbial water-quality thresholds, standardized sampling and testing procedures, monitoring frequency, maintenance documentation, and reporting criteria for suspected DUWL-associated infections (47).

These indicators could be incorporated into national or regional surveillance systems, allowing standardized microbiological and clinical data to be collected and subsequently aggregated in an anonymized international database (64).

Periodic analysis of these data could identify recurrent contamination patterns, emerging microbial risks, and implementation failures across jurisdictions (28).

An international multidisciplinary working group could then use these findings, together with emerging scientific evidence, to periodically update a core set of evidence-based recommendations. Importantly, harmonization should focus on common minimum safety outcomes and surveillance principles rather than identical operational requirements, thereby allowing individual jurisdictions to adapt implementation strategies to their regulatory structures, available resources, and dental-unit technologies (64).

### 5.6 Limitations

This review has several limitations that should be considered when interpreting its findings. First, the literature search was restricted to publications and official guidance documents available in English, which may have resulted in the omission of relevant regulations published exclusively in other languages. To minimize this limitation, official English versions were used whenever available. For jurisdictions where English versions were unavailable, the original regulatory documents were translated by the authors to facilitate data extraction and comparison.

Second, the structured database search was limited to publications published between January 2020 and November 2025 to capture contemporary evidence and current regulatory recommendations. However, seminal publications, historical guidance documents, and foundational references published before this period were identified through targeted hand-searching of reference lists and official regulatory sources to provide historical context and ensure a comprehensive overview of the evolution of DUWL management.

Third, because this study was conducted as a narrative review, a formal methodological quality appraisal or risk-of-bias assessment was not performed, and quantitative evidence synthesis or meta-analysis was beyond the scope of this review due to the heterogeneity of the included evidence.

Finally, this review intentionally integrates evidence from peer-reviewed publications, national and regional regulations, professional guidelines, manufacturer instructions, and other grey literature to provide a comprehensive understanding of current DUWL management practices. While this broad approach represents a major strength of the review, differences in document type, reporting standards, regulatory frameworks, and the frequency with which guidance documents are updated limited direct comparisons across jurisdictions. Although every effort was made to provide an accurate and balanced synthesis of the available evidence, some degree of interpretative subjectivity is inherent in reviews of this nature.

## 6 Conclusion

DUWL contamination continues to represent a critical yet frequently underestimated dimension of infection prevention, with implications that extend well beyond the dental operatory. When viewed through the lens of the One Health approach, DUWLs emerge as interconnected environmental reservoirs that influence not only patient outcomes but also broader efforts to contain AMR. From a One Health perspective, improving DUWL management requires an integrated approach that recognizes the interdependence of environmental water quality, microbial ecology, human health, and antimicrobial stewardship. Strengthening international surveillance and harmonizing evidence-based guidance will be important steps toward addressing these interconnected challenges.

As new technologies continue to develop, improving DUWL management will rely on finding practical and scalable ways to apply them in clinical settings on a global scale. Emerging tools, ranging from real-time microbial sensing and AI-driven risk prediction to advanced antimicrobial materials, offer opportunities to redefine how DUWL safety is maintained and monitored. The development of future international recommendations would also benefit from closer collaboration among regulatory authorities, professional organizations, researchers, and dental unit manufacturers to ensure that evidence-based guidance is both scientifically robust and practically applicable across different healthcare settings. Equally essential is a sustained commitment to increasing DUWL awareness in profession through modernized curricula, competency-based training, and ongoing education. Ultimately, improving the safety of dental treatment water extends beyond guideline development and technical solutions. It requires a systems-level commitment to harmonized policies, evidence-based implementation, routine monitoring, international collaboration, continuous innovation, and sustained professional engagement to protect patients, oral healthcare professionals, and the broader healthcare system.
