# Supplementary material for: Global variability in dental unit waterline safety standards: bridging the gap between guidelines and practice
Source: Front Oral Health. 2026 Oct 14;7:1896545. doi: 10.3389/froh.2026.1896545 (PMC13714496; doi:10.3389/froh.2026.1896545)
Supplement: Supplementary file 1 [file Table1.docx]

| Database | Search strategy | Date |
| --- | --- | --- |
| Pubmed | ( "dental unit waterline*"[Title/Abstract] OR "dental waterline*"[Title/Abstract] OR DUWL[Title/Abstract] ) AND ( biofilm[Title/Abstract] OR "microbial contamination"[Title/Abstract] OR "water quality"[Title/Abstract] ) AND ( disinfection[Title/Abstract] OR cleaning[Title/Abstract] OR flushing[Title/Abstract] OR monitoring[Title/Abstract] ) AND ("2020/01/01"[Date - Publication] : "2025/11/30"[Date - Publication]) | 20 Nov 2025 |
| scopus | TITLE-ABS-KEY ( ( ( "dental unit waterline*" OR "dental waterline*" ) AND ( biofilm OR "water quality" OR disinfection OR cleaning OR monitoring OR guideline* OR recommendation* ) ) AND PUBYEAR > 2019 ) | 20 Nov 2025 |
| Web Of Science | ( ( "dental unit waterline*" OR "dental waterline*" ) AND ( biofilm OR Legionella OR Pseudomonas ) AND ( disinfection OR cleaning ) NOT ( endodont* OR wastewater ) ) | 20 Nov 2025 |
